# Altered DNA repair; an early pathogenic pathway in Alzheimer’s disease and obesity

**DOI:** 10.1038/s41598-018-23644-4

**Published:** 2018-04-04

**Authors:** Hao Yu, Fiona Edith Harrison, Fen Xia

**Affiliations:** 1 Department of Radiation Oncology, University of Arkansas for Medical Sciences, Little Rock, AR 72205 USA; 2 Department of Medicine, Vanderbilt University Medical Center, Nashville, Tennessee 37232 USA

## Abstract

Unrepaired DNA double-strand breaks (DSBs) are lethal. The present study compared the extent of DSBs, neuronal apoptosis, and status of two major DSB repair pathways - homologous combinational repair (HR) and nonhomologous end-joining (NHEJ) - in hippocampus of 5–6 month and 16–18 month-old wild-type and APP/PSEN1 mice fed control diet or high fat diet (60% fat from lard). We performed immunohistochemical staining and quantification for nuclear foci formation of γ-H2AX for DSBs, RAD51, and 53BP1, which represent the functional status of HR and NHEJ, respectively. Increased γ-H2AX and caspase-3 staining indicated greater DSBs and associated neuronal apoptosis in APP/PSEN1 mice at both ages studied. RAD51-positive foci were fewer in APP/PSEN1 indicating that HR processes may be diminished in these mice, although NHEJ (53BP1 staining) appeared unchanged. High fat diet in young wild-type mice led to similar changes to those observed in APP/PSEN1 mice (γ-H2AX and caspase-3 staining, and fewer RAD51-positive foci). Overall, these data suggest that APP/PSEN1- and high fat diet-associated early accumulation of DSBs and neuronal cell death, resulted at least in part, from inhibition of HR, one of the major DSB repair pathways.

## Introduction

The typical pathological hallmarks of Alzheimer’s disease include accumulation of β-amyloid, tau hyperphosphorylation, synaptic damage, and cell death. Nevertheless, heterogeneity in Alzheimer’s populations, and failure of β-amyloid-targeted drug trials signal a need to investigate alternative targetable-pathways that may occur upstream in the disease process. Several new lines of evidence indicate a bidirectional relationship between these common pathological changes observed in Alzheimer’s disease, and DNA damage^1,2^. High levels of oxidative damage to DNA (as indicated by 8-OHdG and γ-H2AX) were observed in hippocampal tissue from autopsy in normal aging patients as well as those with neuropathological markers of Alzheimer’s disease, whether with or without dementia^3^. However, patients classified as having neuropathology *with* a dementia diagnosis differed from the two groups with normal cognition, in also showing reduction of markers of DNA repair (PTEN, BRCA1 and p53), and greater TUNEL positive cells indicating cell death. Mre11 complex proteins play a critical role in cellular responses to DNA damage and were substantially reduced in the cortical neurons of Alzheimer’s disease patients compared to age-matched, non-demented controls^4^. Unrepaired, or incorrectly repaired DSBs are the most lethal form of damage that can occur in DNA^5^. Together, these data indicate that failure to repair DNA damage may be the key factor in determining how well the brain is able to adapt to neuropathological damage. The mismatch between patterns of damage and failed repair may be particularly useful in explaining cases where there is discordance between severity of pathology and cognitive deficits. One downstream effect of DSBs, which can be detected histochemically and quantified based on the level of phosphorylation of a histone protein (H2A variant X, at Ser139 (γ-H2AX))^6^, is neuronal death. However, it is still not clear what are the downstream effects of DSBs that can be detected histochemically and quantified, whether these DSB repair mechanisms are altered in Alzheimer’s disease, and at which stage this particular lethal form of DNA damage is involved in the pathologic process^7^.

In the present study we investigated persistent DSBs, and markers of two repair pathways - non-homologous end joining (NHEJ) and homologous recombination-directed repair (HR) - in APP/PSEN1 mice, a model of Alzheimer’s disease-related amyloidosis. These mice exhibit escalating β-amyloid accumulation, oxidative stress, neuroinflammation and cognitive decline from around 6 months of age^8^. We compared mutant and wild-type mice at both this early stage of disease pathogenesis (5–6 months), and as aged animals (16–18 months) when greater neuropathology is observed. We have previously reported that a high fat diet (HFD) led to cognitive deficits in both APP/PSEN1 and wild-type mice, although cognitive decline was greater in the high fat diet-fed transgenic animals^9^. Therefore, a second group of young, wild-type mice was fed a high fat diet for 4 months. We hypothesized that either APP/PSEN1 genotype or HFD would be sufficient to restrict DNA repair efficiency and increase DSBs, and that Nuclear Foci formation of RAD51 or 53BP1 would provide reliable *functional* markers of both HR and NHEJ repair pathways, respectively^10–13^. Our data suggest that these repair process were indeed impacted by both age and genotype, as well as the high fat diet.

## Materials and Methods

### Mice

Female C57Bl/6 J wild-type mice (stock #000664) and male bigenic APP_SWE_/PSEN1_ΔE9_ mice (stock #005864) were obtained from Jackson Laboratories (Bar Harbor, Maine) and used to found the colonies used in this study. All animals were housed in temperature and humidity controlled viviariums and were kept on a 12:12 hour light cycle. All procedures were approved by the Vanderbilt Institutional Animal Care and Use Committee (IACUC). All methods were performed in accordance with IACUC-approved standard operating procedures, or specifically approved procedures, and local regulations Mice were studied at 5–6 months, or 16–18 months. Approximately equal numbers of males and females were used.

### High fat diets

For one cohort of mice, standard lab chow (4% kcal/fat #5001, Purina, St. Louis, Missouri) was replaced from 8 weeks of age with a high-fat diet (HFD; 60% kcal/fat, *#D12492 Research Diets*, New Brunswick, New Jersey). Mice were weighed weekly following the initiation of experimental diets. Mice were euthanized at 5–6 months of age following 16 weeks on the diet.

### Tissue processing

Mice were euthanized by overdose of inhaled isoflurane followed by cervical dislocation. They were then perfused through the heart with 10 mls of saline. Brains were removed quickly, cut along the midline, and immersion fixed in 10% formalin for 72 hours. They were then stored in 10% sucrose at 4 °C until needed. One hemisphere per mouse was paraffin-embedded and sliced on a microtome at a thickness of 4 microns. Sections were mounted, three per slide, for histochemistry. Staining was undertaken using adjacent slides. Sections were selected with reference to a mouse brain atlas^14^ to include hippocampus and overlying cortex.

### β-Amyloid plaque coverage

Thioflavin-S staining was used to confirm the presence of β-amyloid plaques in the APP/PSEN1 mice. Sections were first deparaffinized in two washes of histoclear (xylene substitute, Electron Microscopy Sciences, Hatfield, Pennsylvania) and then rehydrated using a standard ethanol gradient. Thioflavin-S stain (2.5%, Sigma Aldrich, St. Louis, Missouri) was performed with Mayer’s hemotoxylin (Sigma Diagnostics, St. Louis, Missouri) as a counter stain. Images of hippocampus and cortex were taken at 4Xusing EVOSFl microscope and stitched together in photoshop. These composite images were scored for percent plaque coverage of hippocampus and overlying cortex using freely-available software ImageJ (NIH).

### Immunohistochemical techniques

Brain sections were deparaffinized as above, rehydrated, and then incubated in 3% hydrogen peroxide for 20 minutes to quench endogenous peroxidase activity. The sections were fixed in 4% paraformaldehyde (PFA) for 20 minutes then washed with PBS + 0.1% Triton three times, before incubation with 10% normal goat serum in PBS + 0.1% Triton for 30 min to block the background. Incubation with i) NeuN with γ-H2AX, ii) NeuN with Rad51, iii) NeuN with 53bp1, or iv) NeuN with Caspase-3 were performed as described elsewhere^15,16^. Mouse anti-NeuN (Abcam, Cambridge, Massachusetts), Rabbit anti-Caspase3 (Cell signaling, Danvers, Massachusetts), Rabbit anti-Rad51 (Abcam, Cambridge, Massachusetts), Rabbit anti-53bp1 (Millipore, Billerica, Massachusetts) and Rabbit anti-phospho-γ-H2AX antibody (Abcam, Cambridge, Massachusetts) were used as the primary antibodies, each at 1:500 dilution in PBS + 0.1% Triton + 1% normal goat serum at 4 °C overnight. Secondary antibodies used were 1:1000 anti-rabbit Alexa488 and anti-mouse Alexa594 conjugated antibodies (Invitrogen, Carlsbad, California). The sections were mounted using ProLong Gold with DAPI (Invitrogen, Carlsbad, California) for imaging.

For each animal, the cells were counted in sections from the anterior hippocampus (equivalent adult mouse interaural coordinates 2.10 to 1.98 mm) in CA1, CA2, CA3 (not shown) and DG^17^. Total cell numbers for each section were counted under a fluorescent microscope (original magnification, ×40; Carl Zeiss, Thornwood, New York). Cells were counted in at least three slides per mouse, with three to six mice per group. Rad51 foci should only be detectable in new neurons and not mature or differentiated cells, Rad51 foci are calculated relative to area (calculated as mm^2^) and not number of NeuN-positive cells. Experimenter was blinded to animal genotype or treatment during histology imaging and cell counting.

### Statistical analyses

Data were analyzed using Prism version 6. All data are reported as group means, with error bars reflected the standard error. Individual data points are shown for clarity. The number of stain-positive caspase 3, and the foci of H2AX and 53BP1 were calculated as a percent of NeuN positive cells, except for RAD51 which are calculated based on area. Differences according to genotype were analyzed by two-tailed t-test relative to wild-type age- and diet-matched controls. Additional comparisons were made between age-matched wild-type mice on control versus high fat diet. We did not make a direct comparison according to age, nevertheless to decrease the potential for type 1 error, P values for comparisons in Experiment 1 (2 ages) were accepted as significant at 0.025 to correct for separate comparisons in the two age groups.

## Results

**Figure 1:**
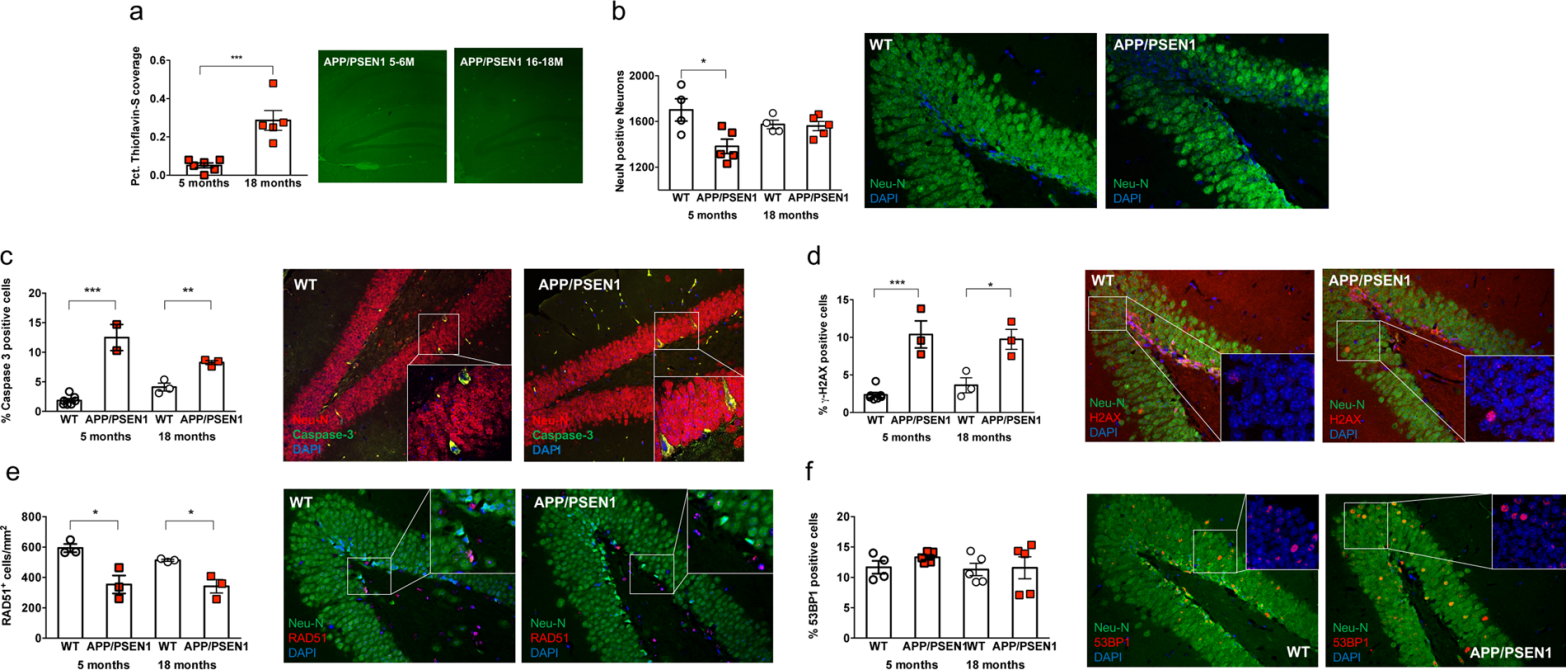
Increased unrepaired DNA damage in hippocampus in young and aged APP/PSEN1 mice. (**a**) β-amyloid plaques were detectable in low numbers, and small sizes in young APP/PSEN1 mice, but percent coverage increased significantly in older mice (t(9) = 4.80, p < 0.001). (**b**) Total neuron number (NeuN-positive cells) in hippocampus was lower in young APP/PSEN1 mice (red squares) compared to wild-type (WT, open circles) litter-mates (t(7) = 2.865, p = 0.024). (**c**) Apoptosis, as measured by percent Caspase 3-positive neurons was elevated in APP/PSEN1 mice hippocampus at both ages (t(7) = 9.52, p < 0.001; t(4) = 5.57, p = 0.0051). (**d**) Persistent DNA damage (γ-H2AX-staining of DSB) increased in the APP/PSEN1 mice at both ages (t(8) = 6.79, p < 0.0001; t(4) = 3.672, p = 0.0213). Markers of DNA repair via (**e**) the homologous recombination directed repair pathway (HDR, Rad51, t(4) = 3.667, p = 0.0215) but not through, (**f**) the non-homologous end-joining pathway (NHEJ, 53BP1) were decreased in APP/PSEN1 mice at both time points. (**c–f**). Number of stain-positive caspase 3, and the foci of H2AX and 53BP1 were calculated as a percent of Neu-N positive cells, except for RAD51 which are calculated based on area. Data were analyzed by two-tailed t-test between genotypes, within each age group. *P < 0.025, **P < 0.01, ***P < 0.001 different from wild-types as marked. β-amyloid plaque coverage is shown as difference between ages in APP/PSEN1 mice only ***P < 0.001. All images are taken from dentate gyrus in young mice. Images taken at 10*X*, with inset taken at 40*X* magnification, except for (**a**) which were imaged at 4X.

As expected, a very low level of β-amyloid deposition as plaques was detected in younger APP/PSEN1 mice, but this increased significantly by the older age point (Fig. 1a). Approximately 20% fewer NeuN-positive cells were found in hippocampus of young APP/PSEN1 mice (Fig. 1b) compared to wild-type littermates. These differences were less marked in the aged mice, although more apoptotic cells were still observed in aged APP/PSEN1 mice compared to wild-type (Fig. 1c). We hypothesized that much of this cellular loss may be due to unrepaired lethal DNA DSBs. In keeping with the enhanced caspase-3 staining, APP/PSEN1 mice showed evidence of approximately 3-fold greater persistent DNA DSBs at both ages examined, in the form of γ-H2AX nuclear foci-positive staining, (Fig. 1d). HDR repair processes, indicated by RAD51 nuclear foci positive staining, were diminished in APP/PSEN1 mice compared to wild-type at both ages (Fig. 1e). As a marker of HR, RAD51 is not expected to be observed in post-mitotic cells. RAD51 positive cells were only observed in subgranular zone of dentate gyrus, the site of neurogenesis, in cells that also stained positive for NeuroD (neurogenic differentiation factor 1, neuroprogenitor cell marker, *not shown*). In contrast, NHEJ activity as indicated by 53BP1 nuclear foci positive staining (Fig. 1f), did not differ according to genotype or age.

**Figure 2:**
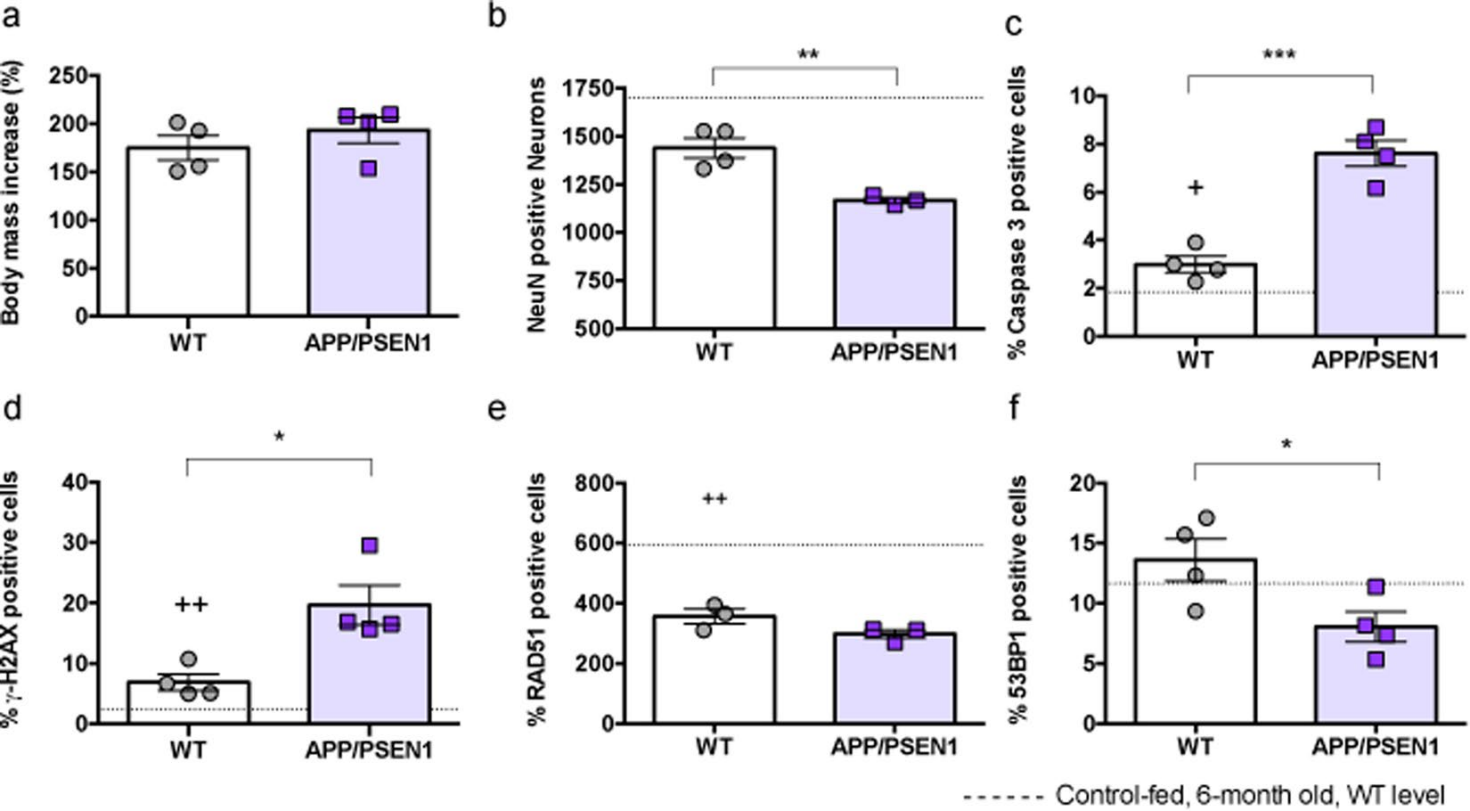
High fat diet (HFD) accelerates DNA damage in 6-month old mouse hippocampus and slows HR repair processes in wild-type and NHEJ APP/PSEN1 mice. (**a**) 16 weeks of HFD-feeding led to equivalent weight gain in both wild-type (WT, grey circles) and APP/PSEN1 (purple squares) mice. (**b**) Fewer NeuN-positive cells were observed in HFD-fed APP/PSEN1 mice compared to wild-types (t(5) = 4.41, p = 0.0069), and a strong trend toward decreased cell count was seen in HFD-fed wild-types compared to controls (dashed line, t(6) = 2.38, p = 0.054). (**c**) Greater apoptosis was seen in HFD-fed APP/PSEN1 mice compared to wild-types (t(6) = 7.236, p = 0.004), and Caspase3-positive cells were increased in HFD-fed wild-types compared to chow-fed controls (t(9) = 2.40, p = 0.040). (**d**) γ-H2AX-foci were more common in HFD-fed APP/PSEN1 (t(6) = 3.59, p = 0.012) compared to HFD wild-type mice, and increased in HFD wild-type mice compared to control wild-types (t(9) = 4.25, p = 0.0021). (**e**) RAD51-positive foci were decreased in HFD-fed wild-type mice compared to controls (t(4) = 6.47, p = 0.0029), but there was no further decrease in APP/PSEN1 mice. (**f**) Fewer 53BP1-positive foci were observed in APP/PSEN1 mice compared to wild-types on HFD (t(6) = 2.59, p = 0.041). Data are analyzed by separate 2-tailed t-tests within each comparison pair. *P < 0.05, ***P < 0.001 all HFD mice, APP/PSEN1 compared to wild-type as marked; ^+^P < 0.05, ^++^P < 0.01 high fat diet-fed wild-type mice compared to age-matched control fed-wild-types (indicated by dashed line).

Given the relationship between obesity, diabetes and Alzheimer’s disease prevalence^18,19^, we sought to determine how 4 months feeding with a high fat diet (HFD, 60% calories from lard) affected DNA damage and repair in young mice. As expected, HFD led to significant weight gain in both genotypes (Fig. 2a). Interestingly, we counted fewer neurons in HFD groups, compared to age-matched, control-fed wild-type mice although the magnitude of difference in the HFD-fed mice appeared to be similar in wild-type and APP/PSEN1 mice (control data from Fig. 1 wild-types represented by dashed lines in Fig. 2b–f for comparison). HFD significantly increased both apoptosis and DNA damage (Caspase 3 and γ-H2AX, Fig. 2c,d) in wild-type mice compared to aged-matched low fat diet controls. Nevertheless, the diet-induced damage was greater in APP/PSEN1 mice. Intriguingly, HFD decreased RAD51 positive foci in wild-type mice to a level observed in control fed APP/PSEN1 mice. No further decrease was observed in the HFD fed APP/PSEN1 group (Fig. 2e). HFD had no further effect on NHEJ repair in wild-type mice, but the combination of diet and genotype led to decreased NHEJ repair in the HFD APP/PSEN1 mice (Fig. 2f).

## Discussion

In this study we report that increases in DNA DSBs (γ-H2AX nuclear foci) and apoptosis (Caspase3) in hippocampal neurons, were accompanied by decreased evidence of HR DSB repair (RAD51) in young and aged APP/PSEN1 mice, and in young mice fed a high fat diet. The novelty of these findings lies in the early time-point that these changes were detected, prior to 6 months of age in the APP/PSEN1 mice, and that similar patterns of damage and failed repair were observed in wild-type mice fed a high fat diet.

The increased apoptosis and cell loss observed in young APP/PSEN1 mice is consistent with previous reports of neuronal loss in these mice^20,21^, and likely reflects a combination of changes in apoptosis rates, DNA damage and failed DNA repair. Overall, differences in neuronal populations should be considered in terms of the balance between cell death and neurogenesis, the latter of which was not assessed in this study. Nevertheless, the increased γ-H2AX-positive staining recapitulated data from 6-month old J20 human APP expressing line (J20)^7^. DSBs can be generated by normal physiological activity and are efficiently repaired through two major pathways, HDR and NHEJ, often within 24 hours^7^, thus avoiding apoptosis and cell death. RAD51 foci represent on-going repair processes, particularly in proliferating cells. Failure to engage the DNA repair systems and initiate repair, as reported here, is therefore likely a key mechanism for determining early damage in Alzheimer’s disease. The increase in DSBs from HFD feeding and Alzheimer’s disease mutations, combined with the already-diminished HR repair capacity in mutant mice, and additional decrease in NHEJ repair, could explain, at least in part, the accelerated pathological process toward cognitive decline we have previously observed in HFD-fed APP/PSEN1 mice^9^.

HDR takes place only in S-phase of proliferating and undifferentiated neuronal stem and progenitor cells. NHEJ can occur in both proliferating and differentiated neuronal cells, and is the prevalent DSB repair pathway in post-mitotic neurons, although it is prone to generation of more errors than HDR^5^. NHEJ was decreased in nuclear cortical extracts from brains of Alzheimer’s disease versus normal control subjects^22^. In our study, not only was NHEJ (53BP1) not upregulated in the young or aged APP/PSEN1 mice despite greater DSBs (γ-H2AX), it was downregulated in the obese APP/PSEN1 mice indicating a likely pathway for the resulting greater damage. DNA damage (not specifically DSBs) is induced within hours in cultured cortical neurons following addition of β-amyloid^7^, whereas apoptotic markers were not observed in the cells until 48 hours later^23^. Even low, sub-lethal concentrations of aggregated β-amyloid_25–35_ decreased DNA DSB repair pathways *in vitro* in PC12 cells. γH2AX/53BP1-positive foci were observed in hippocampi of J20-hAPP mice by 1.5 months, prior to any significant amyloid-β accumulation^7^. Genomic instability, included failures in DNA repair, is likewise thought to contribute to pathogenesis as a consequence of C9orf72 repeat expansions (a noncoding region of chromosome 9) in amyotrophic lateral sclerosis and a number of other degenerative diseases including Alzheimer’s disease^24,25^. Further, mice carrying a neuron specific mutation in Ercc1 (excision repair cross-complementing group 1, knockout), have impaired DNA repair, including of DSBs, and show both DNA damage and neurodegeneration, and age-dependent cognitive decline and by 6-months. It is therefore extremely difficult to separate the different factors of β-amyloid, and other pathologies including inflammatory response, which each drive localized generation of reactive oxygen species^26^. It is likely that some combination of each of these acting synergistically contribute to the patterns of damage and failed repair observed. Surprisingly, the extent of differences in DNA damage and repair pathways between wild-type and APP/PSEN1 mice were similar at both ages, supporting a β-amyloid-independent mechanism. Although the data reported here do not explain the mechanism behind the shared deficiency in HR-repair processes in the APP/PSEN1 and HFD-fed mice, they illustrate a novel phenotype that will bear closer study.

Changes specific to the dentate gyrus typically reflect the processes that occur in normal aging, whereas changes localized elsewhere, spreading from temporal cortex and throughout hippocampus are more reflective of the pattern of damage observed in Alzheimer’s disease. We observed similar patterns of staining in the dentate gyrus as in the rest of the hippocampus (*data not shown*), indicating that the pattern of DNA damage we observed is likely more closely related to pathogenic changes relating to disease, rather than normal aging-related changes.

We chose to study damage in tissue slices in the present study because they provide an illustrative snapshot of the brain at a given age. These stains are more specific than, for example, widely used COMET assays, although they do not provide the same level of kinetic information that can be obtained from primary culture models. In the present situation it is not possible to accurately model *acutely* with DNA repair kinetic experiments, the changes that may occur in the *chronic* conditions tested in the mice. For example, the APP/PSEN1 model not only has accumulating amyloid, but also oxidative damage, neurotransmitter changes and altered glial response and responsiveness. Modeling response to high fat diet in culture is also limited by the multiplicity of changes including neuroinflammation, glucose metabolism changes, and other lipid dietary components. Nevertheless, future work must now be conducted to assess in more detail the balance of changes in DNA damage and repair mechanisms in Alzheimer’s disease- and obesity- related models including analysis of a larger group of repair proteins.

Although increased DSBs have previously been observed in other mouse models of amyloidosis, it is notable that dietary fat intake in young mice of both genotypes, created an array of DNA damage and failed repair that resembled that of aged APP/PSEN1 mice. In fact, young wild-type mice fed the high fat diet showed a similar pattern of increased damage, and potentially more devastating failed repair, than was seen in young APP/PSEN1 mice on the control diet. Long term feeding with the same high fat diet used in the present study accelerated cognitive decline in APP/PSEN1 mice to a greater extent than wild-type littermates, and increased β-amyloid accumulation, tau phosphorylation and inflammatory response^9^. Long term high fat diet has also been shown to upregulate APP gene expression^27^ and induce amyloid deposits even wild-type mice, in combination with glial activation^28^. Other studies in APP/PSEN1 mutants report high fat feeding elevates inflammatory response and oxidative stress without altering amyloid deposition^29^. The similar pathways in both obese young and transgenic mice suggest that this pathway may be important in normal and pathological aging. The detrimental effects of HFD observed in wild-type mice (increased apoptosis and DSBs), as well as changes in HR and NHEJ repair pathways have broader, negative implications in non-demented populations in which lifetime obesity rates are now greater than 30%^30^.

The data presented here strongly suggest that alterations in DNA damage and HR repair of DSBs form an important early part of the pathway toward damage and degeneration in Alzheimer’s disease^31,32^ and indicate that similar patterns of damage can be triggered by obesity. Learning and memory in APP/PSEN1 mice are similar to wild-type littermates at this age (up to 6 months), except when additional stressors such as dietary change accelerate cognitive decline and neuropathological damage^9,33^. Given that DNA damage and repair changes were observed in young mice (<6 months of age), it might also be pertinent to assess the severity and persistence of early life HFD on possible epigenome-wide changes to related genes and to discover how these relate to cognitive function. In particular, specific pathways relating to Alzheimer’s disease, or other neurodegenerative disease, may be differentially regulated in obese and normal weight conditions. Furthermore, although we have restricted our studies to neural tissue it might also be of interest to study changes in DNA repair in other organs and tissue types in obesity (for example in adipose tissue itself). Clarifying any such pathways could highlight potentially targetable areas and timelines for intervention. It is now important to identify the extent to which DNA damage contributes to cognitive decline in Alzheimer’s disease, and whether the converse is also true, that Alzheimer’s neuropathology drives DNA damage^34,35^.
